# Minimally Invasive Techniques to Avoid Sternotomy in Complex Lead Extraction Cases

**DOI:** 10.19102/icrm.2019.100201

**Published:** 2019-02-15

**Authors:** Ryan Azarrafiy, Roger G. Carrillo,

**Affiliations:** ^1^University of Miami Miller School of Medicine, Miami, FL, USA; ^2^The Heart Institute at Palmetto General Hospital, Hialeah, FL, USA

**Keywords:** Cardiac implantable electronic device, minimally invasive, pacing, surgery, transvenous lead extraction

## Abstract

Cardiac device lead extractions have increased in frequency over the past several years. Although most of these procedures are successfully performed through a percutaneous approach, certain cases may be unmanageable using conventional methods. The traditional approach for such complex cases has been median sternotomy. However, four surgical techniques offer a less-invasive alternative. These include the transatrial approach, the subxiphoid approach, the left minithoracotomy/thoracoscopy, and the ministernotomy. In the present study, we reviewed data from patients who underwent minimally invasive, surgical lead extraction at our institution from January 2003 to October 2017 using an ongoing, prospective registry. Summary statistics were generated for age, sex, device extracted, lead dwell time (years), procedure indication, major/minor complications and procedural success as defined by the 2017 Heart Rhythm Society consensus statement, and survival at discharge. Between January 2003 and October 2017, 14 cases at our center were managed via a transatrial approach, whereas 11 involved the subxiphoid approach, 19 involved a left minithoracotomy or thoracoscopy, and one involved a ministernotomy. For the transatrial approach, all cases were classified as procedural successes and all patients were discharged alive. Additionally, for the subxiphoid approach, all cases were deemed procedural successes, whereas survival at discharge was 90.9%. For the left minithoracotomy/thoracoscopy, all cases were procedural successes and survival at discharge was 94.7%. Lastly, the ministernotomy was successfully used to remove an infected, retained lead fragment from the innominate vein. In conclusion, at our institution, the transatrial approach, the subxiphoid approach, the left minithoracotomy/thoracoscopy, and the ministernotomy were used as minimally invasive, surgical approaches that represent fairly safe and effective alternatives to median sternotomy in complex cases unamenable to management via conventional, percutaneous approaches to lead extraction.

## Introduction

**Figure 1:**
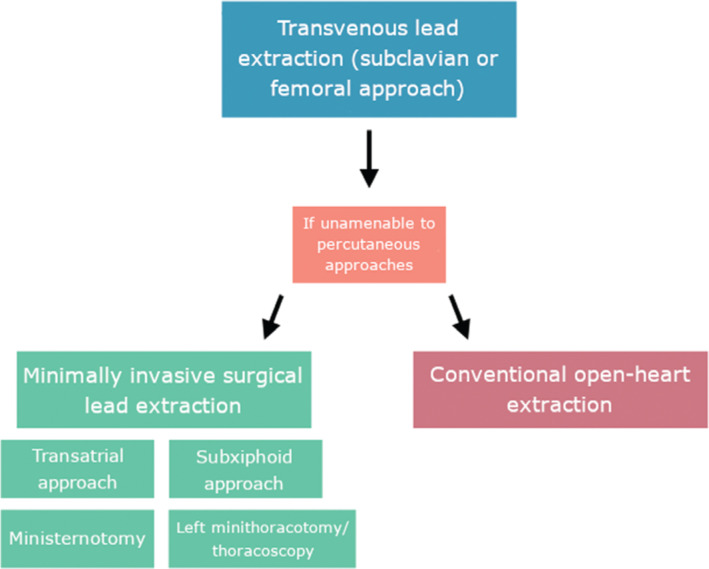
Flowchart of minimally invasive approaches to lead extraction.

Cardiac implantable electronic devices (CIEDs) represent a defined cornerstone of medical practice, as millions of patients worldwide continue to demonstrate cardiac arrhythmias requiring treatment with these devices.^1^ Yet, in rare circumstances, CIEDs can become infected or malfunction. In these cases, removal of both the device generator and its associated leads is indicated. In a procedure called lead extraction, device leads implanted for more than one year are removed with specialized equipment, often through transvenous use of a laser-powered sheath. Lead extraction has been proven to be a safe and effective procedure that is performed almost exclusively through a percutaneous approach. In this operation, electrophysiologists choose to perform the extraction through the subclavian vein, or, in more complex cases, through the femoral vein.^2^ However, certain complex cases may be unamenable to either of these percutaneous techniques, requiring a surgical approach. Although the traditional approach to complex cases unamenable to percutaneous extraction has been the performance of a median sternotomy and open removal of device leads, four surgical approaches exist that are less invasive. These include the transatrial approach, the subxiphoid approach, the left minithoracotomy/thoracoscopy, and the ministernotomy **(Figure 1)**.

In this article, we explain and assess our institution’s experience with these four minimally invasive, surgical approaches to lead extraction.

## Methods

### Indications and techniques for minimally invasive procedures

#### Transatrial approach

The transatrial approach involves a minimally invasive incision to remove leads directly from the right atrium under fluoroscopic guidance. This procedure offers an alternative to median sternotomy for leads that have perforated the right atrium or lead fragments that have been abandoned and that cannot be retrieved by conventional, transvenous techniques.

**Figure 2:**
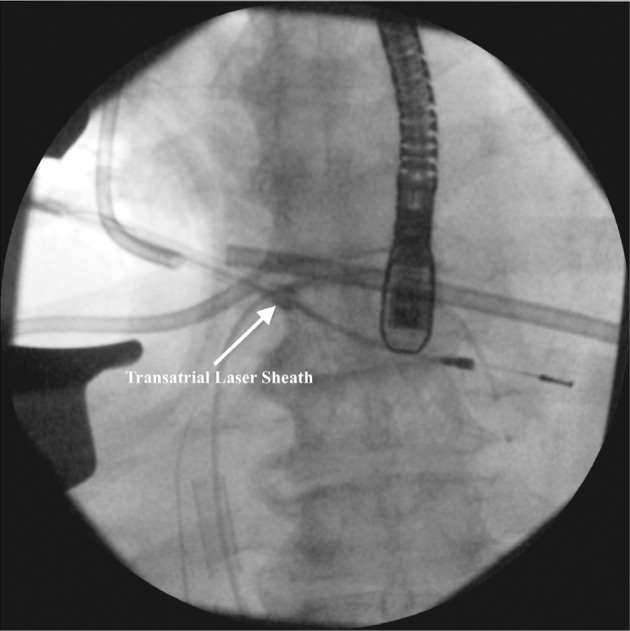
Fluoroscopic image of lead extraction through a transatrial approach.

In the transatrial approach, our standard anesthetic protocol is followed, which includes placing the patient under general anesthesia in a supine position and obtaining arterial lines for blood pressure monitoring and large venous lines for volume resuscitation, respectively. The patient is then intubated with a single-lumen endotracheal tube including a right bronchial blocker. A small, two-inch (5-cm) incision is made at the level of the fourth intercostal space from the midclavicular line to the anterior axillary line. The ribs and pleura are dissected and a longitudinal two-inch (5-cm) incision is used to open the pericardium anteriorly to the phrenic nerve. Next, a purse string is placed on the right atrium. The lung is collapsed and, under fluoroscopic guidance, the lead is retrieved and pulled out of the right atrium using a rongeur. Laser or mechanical tools are used to remove the leads from the heart in an antegrade or retrograde direction. The extraction sheath is advanced in the direction of either the apex of the heart or the subclavian vein, depending on the segment that must be removed. The whole procedure can be done with or without the aid of an endoscope. The actual extraction process is guided with fluoroscopy **(Figure 2)**. Following the extraction of the lead(s), an 18-French (Fr) chest tube is placed and removed 24 hours after the procedure.

#### Subxiphoid approach

The subxiphoid approach involves making an incision below the xiphoid process to extract leads that have chronically perforated the right ventricle or the vestibule of the right atrium. Furthermore, this technique is generally used to extract epicardial leads.

In the subxiphoid approach, our standard anesthetic protocol is followed. A two-inch (5-cm) incision is made just below the xiphoid process and a retractor is used to lift the sternum upward. The pericardium is opened and the leads are visualized. The leads may be extracardiac or epicardial. If they are extracardiac, the protruding portion may be removed from the subxiphoid approach. First, a purse string is placed and then the distal end of the lead is cut at the level of the myocardium. The lead will retract into the ventricle. The purse string is tied and the more proximal portions of the lead can then be removed through a standard percutaneous approach. For epicardial leads, dissection with electrocautery at a low-voltage setting can be helpful and at times may be assisted by fluoroscopy. Following lead removal, hemostasis is obtained, a 10-mm flat drain is placed in the pericardial space before skin closure, and the small incision is closed. The drain is removed 24 hours following the procedure.

#### Left minithoracotomy/thoracoscopy

If there is an extracardiac lead located more toward the apex or left side of the heart, then the left minithoracotomy or thoracoscopy option can be utilized. Moreover, this approach can be used for leads that have perforated the coronary sinus or leads located in the epicardium of the left ventricle.

**Figure 3:**
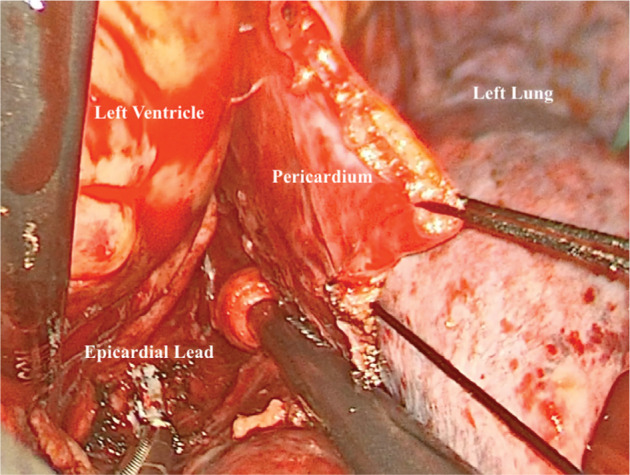
Endoscopic view of epicardial lead extraction via a left thoracoscopy approach.

In the left minithoracotomy/thoracoscopy approach, our standard anesthetic protocol is followed in addition to using a left bronchial blocker to collapse the lung. Fluoroscopy is first employed to locate the exact location of leads in order to determine where an incision is to be made. Next, a small incision is made in the intercostal space, which varies based on the exact location(s) of leads as determined by fluoroscopy. The ribs, pleura, and pericardium are dissected anteriorly to the phrenic nerve. If the lead has perforated the coronary sinus, the protruding portion may be removed by way of the left minithoracotomy/thoracoscopy approach. First, a purse string is placed and then the distal end of the lead is cut at the level of the myocardium. The lead will retract into the ventricle. The purse string is tied, and the more proximal portions of the lead can then be removed through a standard percutaneous approach. If the lead being extracted is epicardial, an endoscope can be used to guide the dissection process with electrocautery at a low-voltage setting **(Figure 3)**. An 18-Fr chest tube is placed prior to skin closure and subsequently removed 24 hours after the procedure.

#### Ministernotomy

The ministernotomy is a rare procedure used predominantly in patients with retained lead segments in the innominate vein that could not be removed via other methods.

In the ministernotomy procedure, our standard anesthetic protocol is followed. Then, the lead fragment is located under fluoroscopy. The manubrium is cut from the jugular notch up to the second intercostal space with a small, sternal saw. A retractor is used to open the manubrium. Proximal and distal control of the innominate vein are obtained. The vein is then opened, the fragment is removed, and a direct or patch repair is performed to close the vein. The sternum is reapproximated with a single stainless steel wire. The skin is closed without drainage.

### Data collection

We reviewed data from patients who underwent a minimally invasive, surgical lead extraction at our institution from January 2003 to October 2017 using an ongoing, prospective registry. Summary statistics were generated for age, sex, device extracted, lead dwell time (years), procedure indication, major/minor complications and procedural success as defined by the 2017 Heart Rhythm Society Consensus, and survival at discharge. All statistics were generated using JMP Pro 13 (SAS Institute, Cary, NC, USA).

## Results

Out of 1,480 lead extractions performed at our center from January 2003 to October 2017, 45 cases (3%) involved a minimally invasive, surgical approach.

### Transatrial approach

At our center, 14 cases employed a transatrial approach between January 2003 and October 2017 **(Table 1)**. The average age for patients undergoing the procedure was 64.14 years ± 20.9 years. Eleven patients were male (78.6%) and three patients were female (21.4%). The devices extracted were as follows: eight pacemakers (57.2%), five implantable cardioverter-defibrillators (ICDs) (35.7%), and one cardiac resynchronization therapy defibrillator (CRT-D) (7.1%). The average lead dwell time was 10.5 years ± 6.96 years. Eleven devices were extracted due to infection (78.6%), whereas the remaining three were extracted because of malfunction (21.4%). Out of the 14 cases that utilized the transatrial approach, none had major complications. One minor complication was observed involving a small pocket hematoma. All cases were a procedural success, and all patients were discharged alive.

### Subxiphoid approach

Our center performed a total of 11 subxiphoid extractions from January 2003 to October 2017 **(Table 1)**. The average age of these patients was 62.5 years ± 16.6 years. Eight patients were male (72.7%) and three were female (27.3%). Six extracted devices were CRT-Ds (54.5%), three devices were pacemakers (27.3%), and two devices were ICDs (18.2%). In all cases, the patient had epicardial leads. The average lead dwell time was 10.1 years ± 10.3 years. Nine cases required extraction due to infection (81.8%), whereas two extractions were performed due to device malfunction (18.2%). There were no major or minor complications, and all cases were procedural successes. Survival at discharge was 90.9%, as there was one death due to sepsis that occurred unrelated to the procedure.

### Left minithoracotomy/thoracoscopy

Our institution has performed 19 left minithoracotomies or thoracoscopies between January 2003 and October 2017 **(Table 1)**. The average age of these patients was 66.2 years ± 14.3 years. Fourteen patients were male (73.7%) and five were female (26.3%). Eight extracted devices were ICDs (42.1%), seven were CRT-Ds (36.8%), and four were pacemakers (21.1%). The average lead dwell time was 7.15 years ± 6.52 years. Thirteen extractions were due to infection (68.4%) and six were due to malfunction (31.6%). Fourteen cases involved the extraction of epicardial leads (73.7%). There were no major or minor complications, and all cases were procedural successes. Survival at discharge was 94.7%, as there was one death that occurred involving ventricular tachycardia in the postoperative period.

### Ministernotomy

**Figure 4:**
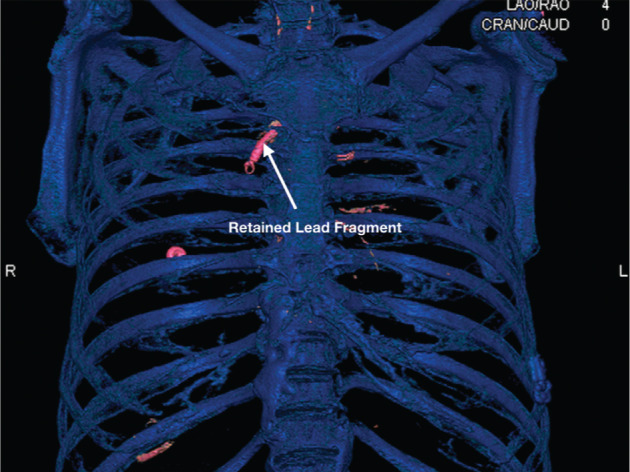
Three-dimensional reconstruction of computed tomography imaging results showing a retained lead fragment in the left innominate vein.

This procedure was performed once at our institution in a 59-year-old male with sepsis and nonischemic, dilated cardiomyopathy who had previously undergone failed transvenous lead extractions at different institutions. Despite numerous attempts to use a percutaneous approach, an infected ICD lead fragment was retained in the left innominate vein **(Figure 4)**. The lead dwell time for the fragment was 7.8 years. Due to the prior failed extractions, the patient was referred to our institution for median sternotomy. However, following a thorough radiologic analysis, a decision was made for the patient to undergo a ministernotomy instead. The procedure was successful, and a subcutaneous ICD was placed one week following the extraction. The patient was discharged with no complications at 12 days following the original procedure.

## Discussion

Surgical lead extraction has evolved significantly over the past several decades. In 1981, Choo et al. reported the open extraction of infected epicardial pacemaker systems.^3^ Furthermore, in 1998, Varma et al. detailed a staged extraction involving a percutaneous laser and an open surgical approach to extract a chronic atrial lead.^4^ In 2010, Rusanov et al. reported a 15-year experience, in which epicardial leads and patches were removed through median sternotomy or a left, right, or subxiphoid thoracotomy.^5^ Yet, more recently, institutions have begun to discuss the use of more minimally invasive, surgical approaches to extract leads as an alternative to median sternotomy or full left or right thoracotomies. For example, in 2012, Curnis et al. described the performance of a left thoracoscopy to complete a failed percutaneous coronary sinus lead extraction.^6^ Over the past several years, several institutions have also described the use of right minithoracotomy, which we have described herein as the “transatrial” approach, to extract fractured leads or in those patients who have been deemed unamenable to a percutaneous extraction.^7–10^ Overall, surgical extraction has advanced significantly over the past several decades, with minimally invasive approaches serving as safe and effective alternatives to median sternotomy in cases that cannot be managed via percutaneous extraction.

At our center, four minimally invasive techniques have been used to provide viable alternatives to median sternotomy with high rates of success. Although all cases at our center resulted in procedural success, mortalities occurred before discharge in the left minithoracotomy/thoracoscopy and the subxiphoid approach groups. However, it is important to note that these deaths occurred in the postoperative period and were unrelated to the procedure. The mortality reported in this cohort encompasses the 30-day mortality rate rather than procedure-related mortality. Furthermore, the patients in this cohort would have otherwise received open sternotomy, exposing them to a greater risk of complications and procedure-related mortality. Thus, minimally invasive approaches represent the preferred alternative in complex cases, offering better outcomes in comparison with open sternotomy.

The advancement of surgical technology has been a major driving force in the transition to these minimally invasive techniques. For example, the development of endoscopic tools has allowed surgeons to visualize thoracic anatomy without the need for an open procedure. Moreover, the intraoperative use of fluoroscopy enables surgeons to localize leads with precision and also guides the deployment of both mechanical and laser extraction tools. Advancements in cardiac anesthesia have also been critical in the development of minimally invasive extraction techniques. As utilized in both the transatrial and left minithoracotomy/thoracoscopy approaches, bronchial blockers enable the selective collapse of a single lung, providing better visualization and control for the operator. Overall, advances in technology have facilitated a transition to minimally invasive procedures, which offer shorter lengths of hospital stay, fewer complications, avoidance of cardiopulmonary bypass, and cosmetically desirable incisions.

In addition to changes in technology, the role of the cardiac surgeon has also evolved in the dynamic field of lead extraction. Before the advent of mechanical and laser tools to remove devices percutaneously, surgical extraction was the only option for patients. Although the cardiac surgeon continues to play a significant role in both the implantation and the extraction of epicardial leads, lead extraction today is done almost exclusively through a percutaneous approach.^11^ Yet, as the literature and our center’s experience have shown, the cardiac surgeon can play a critical role in employing minimally invasive techniques to extract leads in which a percutaneous approach is unfeasible. In addition to this extraction option, the cardiac surgeon must also serve as an essential backup response in the inevitable event of complications during percutaneous procedures. Brunner et al. reported that about 1% of cases at a high-volume extraction center required emergent surgical intervention for complications. Most of these backup interventions required open-chest techniques. However, endovascular techniques, such as the deployment of a cover stent for vascular lacerations, are an emerging consideration.^12^ Such new tools may continue to change the dynamic role of the cardiac surgeon in the field of lead extraction. Regardless of each clinician’s individual role, multiple single-center studies and expert guidelines report that a multidisciplinary approach to lead extraction involving electrophysiologists and cardiac surgeons ensures both safe and successful procedures.^11,13,14^

Overall, several tools and approaches can be employed to safely and effectively manage infected or malfunctioning cardiac devices, and a multidisciplinary decision-making process is critical to a successful procedure. The standard approach to lead extraction is a percutaneous procedure through the subclavian vein with the use of laser or mechanical tools. However, certain extractions cannot be accomplished by way of a subclavian route. As such, alternative percutaneous methods have been successfully described including a femoral or internal jugular approach as well as a combined approach of the two.^15^ However, certain cases remain unamenable to even these percutaneous alternatives, thus often requiring a surgical approach. In the planning stage of lead extraction, a thorough radiologic analysis can help to determine if leads are extracardiac or abandoned. In these cases, a multidisciplinary conversation among electrophysiologists, cardiac surgeons, radiologists, and cardiac anesthesiologists can help to identify cases in which a minimally invasive, surgical lead extraction may be necessary. These procedures should be performed by an operator with significant experience in the surgical management of device leads.

## Conclusion

Several surgical options exist for cases unamenable to conventional, percutaneous approaches to lead extraction. Per the experience at our institution, the transatrial approach, subxiphoid approach, left minithoracotomy/thoracoscopy, and ministernotomy are minimally invasive, surgical approaches that represent safe and effective alternatives to median sternotomy in complex cases.

## Figures and Tables

**Table 1:** Case Information and Demographics for Minimally Invasive Procedures

Approach	Transatrial (n = 14)		Subxiphoid (n = 11)		Left Minithoracotomy (n = 19)		Ministernotomy (n = 1)		All Lead Extractions (n = 1,480)	
Age (years)	64.14 ± 20.9		62.5 ± 16.6		66.2 ± 14.3		59		67.15 ± 15.1	
Sex (% male)	78.6%		72.7%		73.7%		100%		73.3%	
Device(s) extracted	•	5 ICDs (35.7%)	•	2 ICDs (18.2%)	•	8 ICDs (42.1%)	•	1 ICD (100%)	•	624 ICDs (42.2%)
	•	8 PPMs (57.2%)	•	3 PPMs (27.3%)	•	4 PPMs (21.1%)			•	512 PPMs (34.6%)
	•	1 CRT-D (7.1%)	•	6 CRT-Ds (54.5%)	•	7 CRT-Ds (36.8%)			•	332 CRT-Ds (22.4%)
									•	12 CRT-Ps (0.8%)
Lead dwell time (years)	10.5 ± 6.96		10.1 ± 10.3		7.15 ± 6.52		7.8		5.93 ± 5.45	
Indication	•	11 infection (78.6%)	•	9 infection (81.8%)	•	13 infection (68.4%)	•	1 infection (100%)	•	818 infection (55.3%)
	•	3 malfunction (21.4%)	•	2 malfunction (18.2%)	•	6 malfunction (31.6%)			•	582 malfunction (39.3%)
									•	80 other (5.4%)
Prior median sternotomy	2 (14.3%)		1 (9.0%)		5 (26.3%)		0 (0.0%)		455 (30.7%)	
Major complication(s)	0 (0.0%)		0 (0.0%)		0 (0.0%)		No		22 (1.5%)	
Minor complication(s)	1 hematoma (7.1%)		0 (0.0%)		0 (0.0%)		No		79 (5.3%)	
Procedural success	14 (100.0%)		11 (100.0%)		18 (94.7%)		Yes		1,406 (95.2%)	
Survival at discharge	14 (100.0%)		10 (90.9%)		18 (94.7%)		Yes		1,411 (95.4%)	

CRT-D: cardiac resynchronization therapy defibrillator; CRT-P: cardiac resynchronization therapy pacemaker; ICD: implantable cardioverter-defibrillator; PPM: permanent pacemaker.
